# Synthesis and crystal structure of bis­(2-aminobenzimidazolium) *catena*-[metavanadate(V)]

**DOI:** 10.1107/S2056989024005528

**Published:** 2024-06-18

**Authors:** Kholida Jabborova, Jamshid Ashurov, Akmaljon Tojiboev, Shahlo Daminova

**Affiliations:** a Institute of General and Inorganic Chemistry, Academy of Sciences of Uzbekistan, 100170, M. Ulugbek Str 77a, Tashkent, Uzbekistan; b Institute of Bioorganic Chemistry, Academy of Sciences of Uzbekistan, 100125, M. Ulugbek Str 83, Tashkent, Uzbekistan; c University of Geological Sciences, Olimlar Street, 64, Mirzo Ulugbek district, Tashkent, Uzbekistan; d National University of Uzbekistan named after Mirzo Ulugbek University Street 4 Tashkent 100174 Uzbekistan; e Uzbekistan–Japan Innovation Center of Youth, University Street 2B, Tashkent, 100095, Uzbekistan; Vienna University of Technology, Austria

**Keywords:** crystal structure, 2-amino­benzimidazole, hybrid vanadate, hydrogen bonding

## Abstract

The structure of polymeric *catena*-poly[2-amino­benzimidazolium [[dioxidovanadium(V)]-μ-oxido]], {(C_7_H_8_N_3_)_2_[V_2_O_6_]}_*n*_, has monoclinic symmetry. The title compound is of inter­est with respect to anti­cancer activity. In the crystal structure, infinite linear zigzag vanadate (V_2_O_6_)^2−^ chains, constructed from corner-sharing VO_4_ tetra­hedra and that run parallel to the *a* axis, are present. Two different protonated 2-amino­benzimidazole mol­ecules are located between the (V_2_O_6_)^2–^ chains and form classical N—H⋯O hydrogen bonds with the vanadate oxygen atoms, which contribute to the cohesion of the structure.

## Abstract 2

The title compound contains infinite linear zigzag vanadate (V_2_O_6_)^2−^chains, constructed from corner-sharing VO_4_ tetra­hedra, running parallel to the *a* axis.

## 1. Chemical context

In recent years, vanadate compounds have attracted attention in various fields due to their various compositions and inter­esting structures (Smith *et al.*, 2012 ▸; Wutkowski *et al.*, 2009 ▸; Wang *et al.*, 2007 ▸). This is partly due to the ability of vanadium to adopt tetra­hedral [VO_4_], square-pyramidal [VO_5_], trigonal–bipyramidal [VO_5_] or octa­hedral [VO_6_] coordination environments together with possible stable oxidation states of +III, +IV and +V. Inter­estingly, all major vanadate compounds known to date containing cage-, shell-, belt-, barrel-, or basket-shaped entities are structurally related to the layer structure of vanadium pentoxide (Ishaque Khan *et al.*, 2000 ▸). These compounds have many practical pharmacological applications, ranging from anti­cancer agents to anti­fungal agents and, more recently, as insulin mimetics (Singh *et al.*, 2014 ▸; Abakumova *et al.*, 2012 ▸; Amin *et al.*, 2000 ▸) where they inter­act with several points in the cell-signaling pathway associated with the hormone insulin (Amin *et al.*, 2000 ▸; Srivastava & Mehdi, 2005 ▸). Studies have also indicated that vanadate compounds inter­act directly with glucose transporters located on the cell surface (Hiromura *et al.*, 2007 ▸; Makinen & Brady, 2002 ▸). Furthermore, vanadium has been found to have important inter­actions in DNA repair systems, making it a useful target for many oncological/pharmacological studies (Abakumova *et al.*, 2012 ▸; Kostova, 2009 ▸). Given the structural dependence on functions and application, a deeper study of the mol­ecular and crystal structures of such complexes is warranted. In this context, we describe the synthesis and structural features of the polymeric title compound {(C_7_H_8_N_3_)_2_ (V_2_O_6_)}_*n*_.
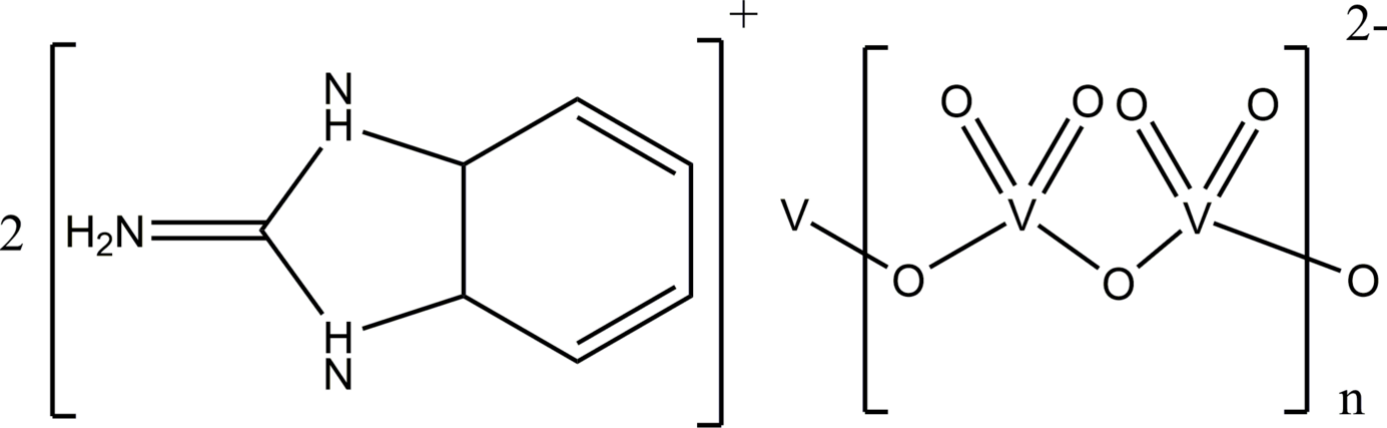


## 2. Structural commentary

**Figure 1:**
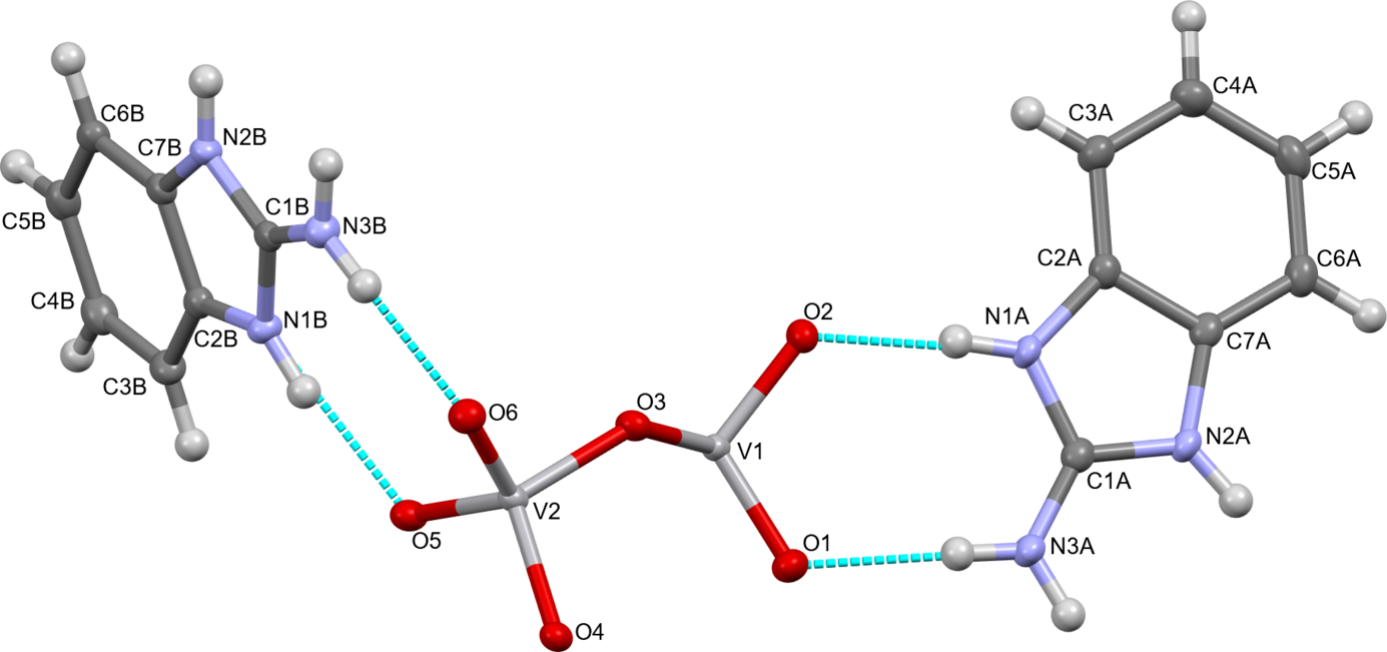
The asymmetric unit of the title compound with the labeling scheme and displacement ellipsoids drawn at the 50% probability level. Dotted lines indicate N—H⋯O hydrogen-bonding inter­actions.

**Figure 2:**
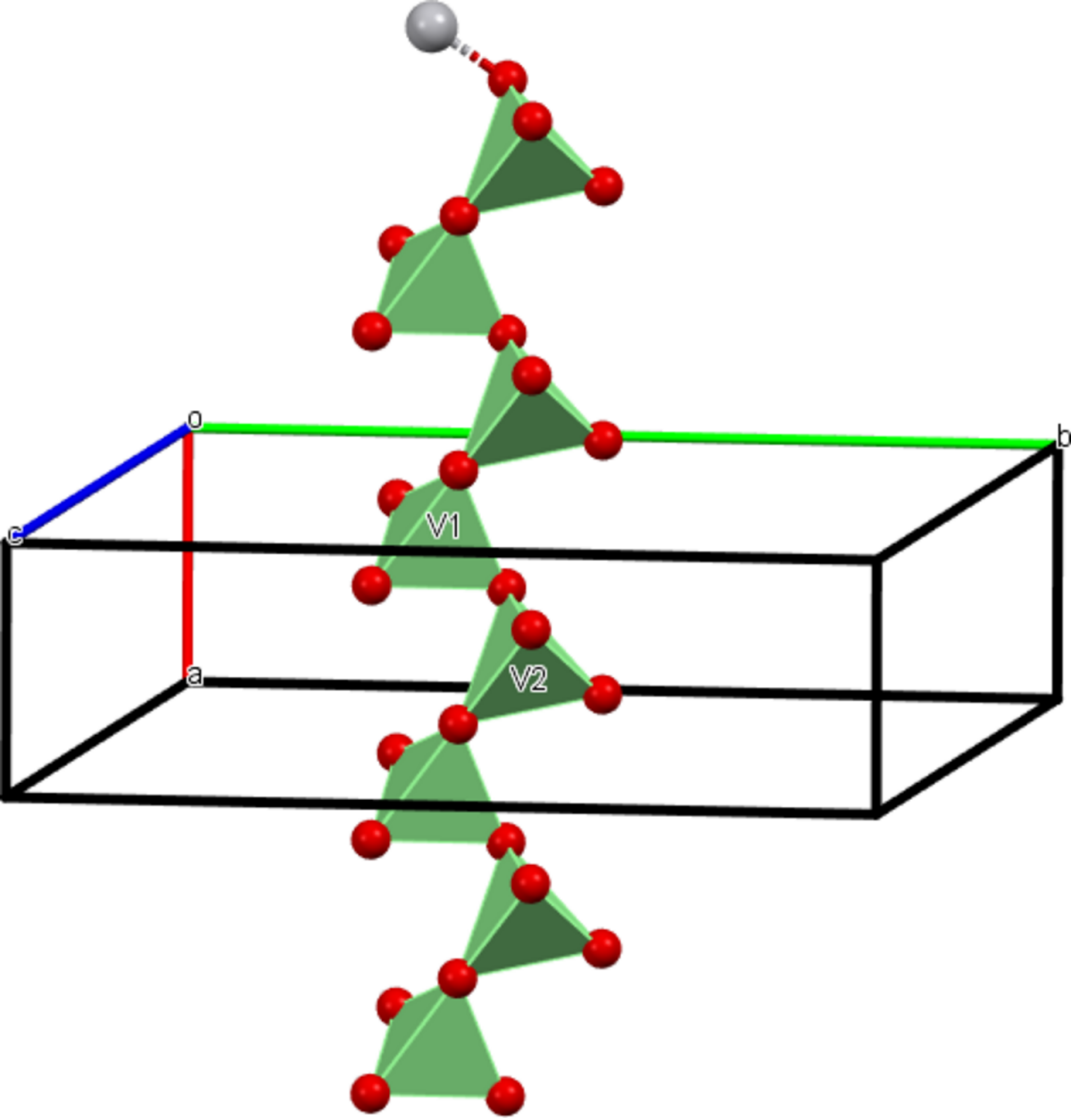
The metavanadate chain with the two different tetra­hedra in polyhedral representation.

The asymmetric unit comprises two 2-amino­benzimidazolium cations (denoted *A* and *B*) and two V and six O atoms of the polymeric metavanadate anion (Fig. 1 ▸). The cationic mol­ecules are almost planar (root-mean-square deviation for *A* = 0.0127 Å and for *B* = 0.0064 Å), and their N—C bond-length distributions are similar to those in related compounds (Aliabadi *et al.*, 2021 ▸; Ruzieva *et al.*, 2022 ▸). The linear zigzag metavanadate (V_2_O_6_)^2–^ chain runs parallel to the *a* axis and is constructed from corner-sharing VO_4_ tetra­hedra (Fig. 2 ▸). Typical for such chains, the bridging O atoms (O3 and O4) have considerably longer V—O bonds than the terminal O atoms (O1 and O2 for the V1O_4_ tetra­hedron and O5 and O6 for the V2O_4_ tetra­hedron; Table 1 ▸). The corresponding V—O and V=O bond lengths are similar to those reported for related hybrid metavanadate compounds (Smith *et al.*, 2012 ▸; Wutkowski *et al.*, 2009 ▸; Wang *et al.*, 2007 ▸; Tyrselova *et al.*, 1996 ▸).

## 3. Supra­molecular features

**Figure 3:**
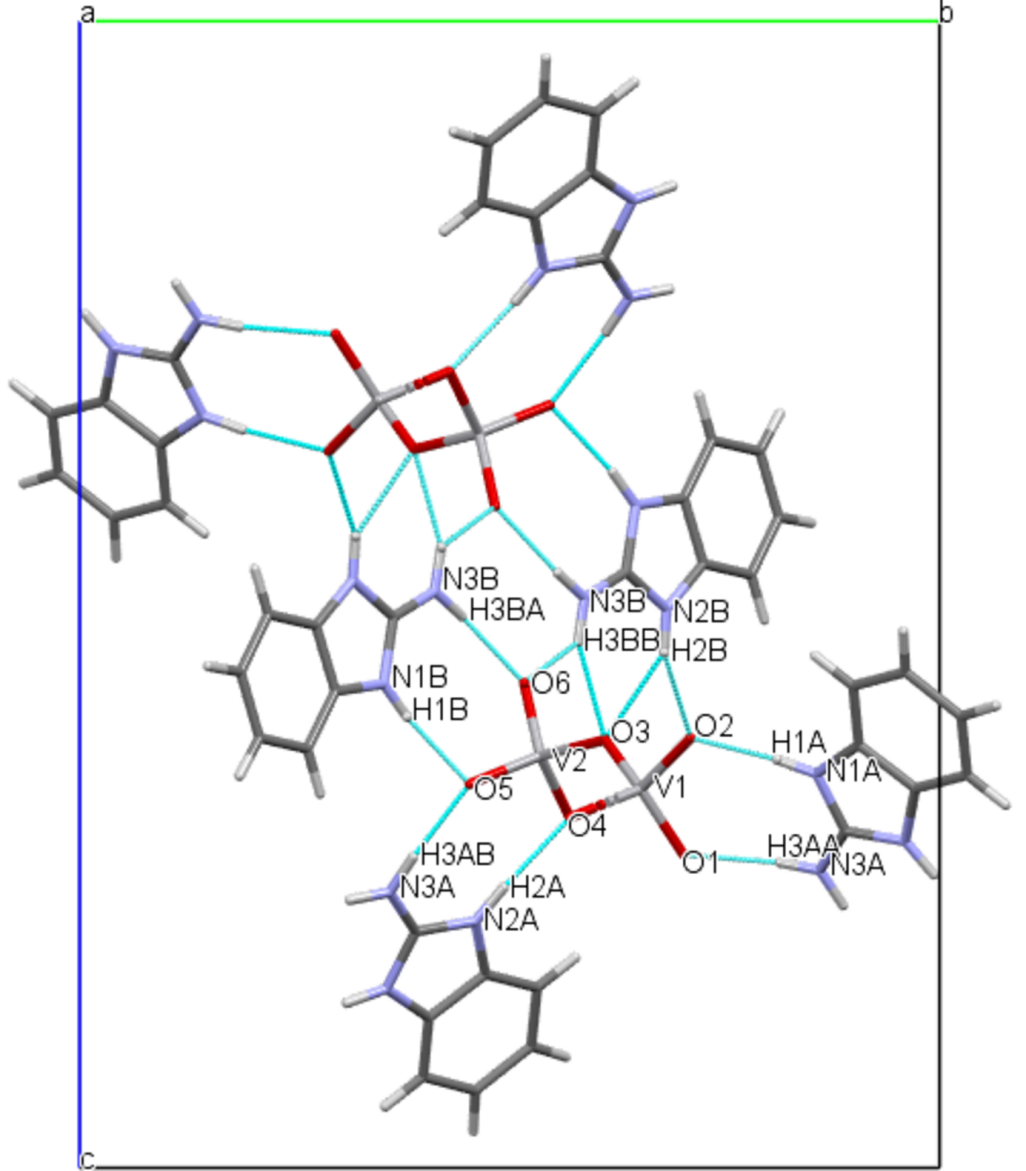
View of the crystal structure of the title compound along the *a* axis, showing N—H⋯O hydrogen bonds drawn as blue dotted lines.

**Figure 4:**
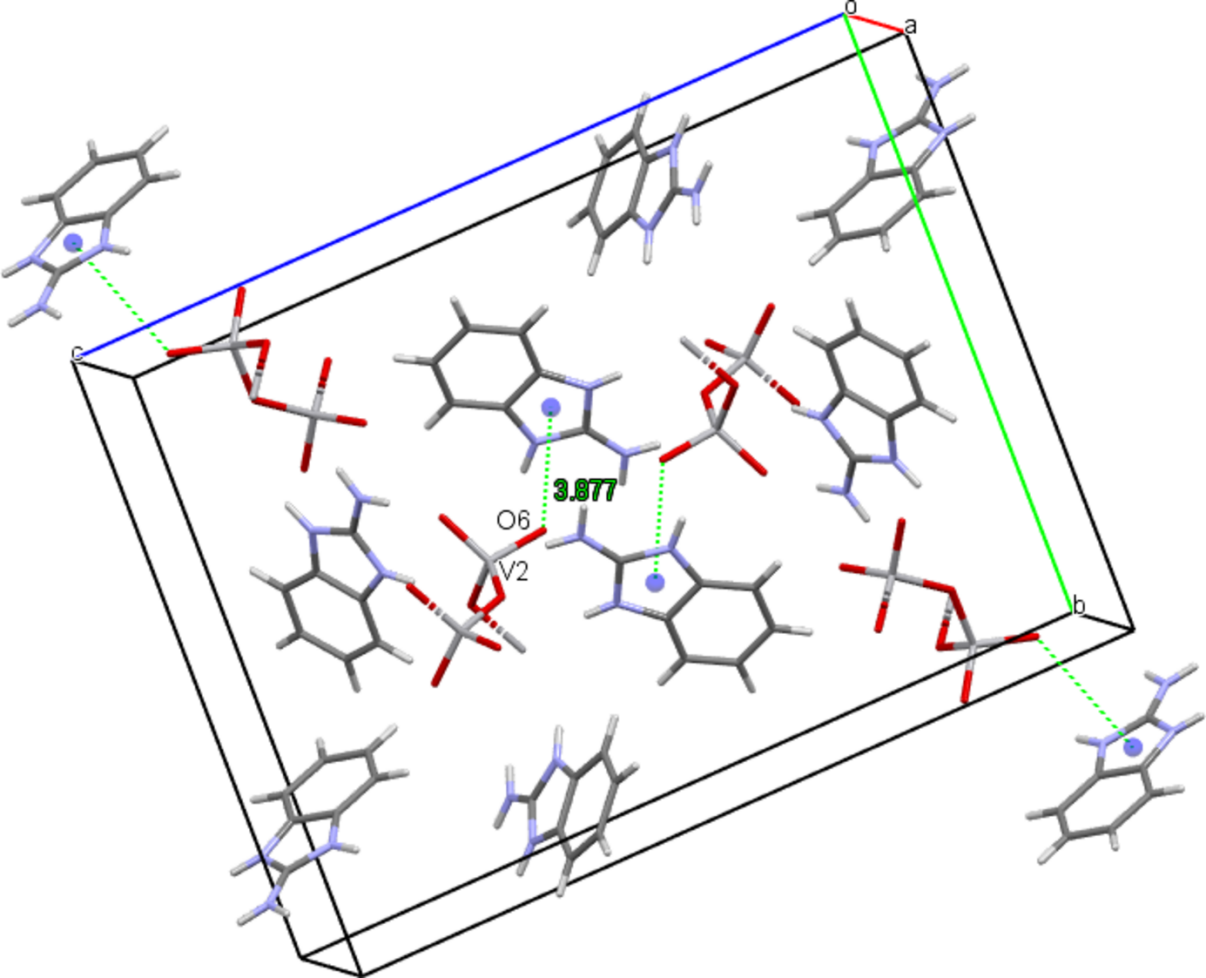
V2—O6⋯*Cg*4 inter­actions in the crystal structure of the title compound.

The crystal packing exhibits an intricate network of classical inter­molecular N—H⋯O hydrogen bonds between the NH and NH_2_ groups of the cations and all oxygen atoms of the metavanadate chain (Fig. 3 ▸, Table 2 ▸). Additional short contacts (Fig. 4 ▸) between the vanadate O6 atom and the centroid of the N1*B*/C1*B*/N2*B*/C7*B*/C2*B* ring (V2—O6⋯*Cg*4(−1 + *x*, *y*, *z*) = 3.8768 (16) Å) consolidate the tri-periodic network structure.

## 4. Database survey

A search in the Cambridge Structural Database (CSD, version 5.43, update of November 2022; Groom *et al.*, 2016 ▸) revealed four hybrid compounds with protonated 2-amino­benzimidazole moieties, two with gallium (WURVAJ, WURVEN; Aliabadi *et al.*, 2021 ▸) and two with lanthanum (JARVOQ, WEGRAF; Ruzieva *et al.*, 2022 ▸). A search for the metavanadate moiety with linear zigzag chains similar to that in the title structure gave the following hits: CEHQEN and CEHQIR, for which dipole moments were calculated using iterative Hirshfeld partial atomic charges (Smith *et al.*, 2012 ▸); (H_3_NCH_2_CH_2_NH_3_)(V_2_O_6_) (FUDLOF02; Ishaque Khan *et al.*, 2000 ▸); 1,6-hexa­nedi­ammonium metavanadate (KOYJAJ; Tyršelová & Pavelčík, 1992 ▸); 3-aza-1,5-penta­methyl­enedi­ammonium metavanadate (KUGGUO; Roman *et al.*, 1992 ▸); [Cu(H_2_O)(C_5_H_14_N_2_)_2_](VO_3_)_2_ (POYNAT; Wutkowski *et al.*, 2009 ▸); *catena*-poly[*N*,*N*′-bis­(2-ammonio­eth­yl)oxamide [dioxidovanadate-μ-oxido-dioxidovanadate-μ-oxido]] (TIGBUH; Wang *et al.*, 2007 ▸); *catena*-poly[2,2′,2′′-nitrilo­tris­(ethanamin­ium) [tri-μ-oxido-tris­[dioxidovanadate(V)]] monohydrate] (VIPRET; Chang *et al.*, 2013 ▸); {piperazinediium poly[trioxo­vanadate], {(C_4_H_12_N_2_)(VO_3_)_2_} (ZITSEA; Tyrselova *et al.*, 1996 ▸); *catena*[bis­[tris­(2-ammonio­eth­yl)amine]­hexa­kis­(μ_2_-oxo)dodeca­oxohexa­vanadium trihydrate] (IMATOG; Li *et al.*, 2009 ▸); *catena*-[penta­kis­(cyclo­hexyl­ammonium) penta­kis­(μ_2_-oxo)deca­oxo­penta­vanadium(V)] (NACFON; Wang *et al.*, 2004 ▸).

## 5. Synthesis and crystallization

All reagents for synthesis and analysis were commercially available and purchased from Sigma Aldrich and used as received without further purification. Chemically pure vanadyl acetyl­acetonate, 2-amino­benzimidazole, and 96% vol ethanol were used. Vanadyl acetyl­acetonate (0.0265 g, 1 mmol) dissolved in 5 ml of EtOH and 2-amino­benzimidazole (0.0133 g, 1 mmol) dissolved in 5 ml of EtOH were mixed with constant stirring until the color of the solution turned to green. The stirring was continued for three hours. The resulting green solution was then allowed to cool to room temperature and green crystals were grown over seven days *via* slow evaporation of the mother liquor. Selected IR bands (KBr pellet, cm^−1^): 3447 (N—H), 1647 (C=N), 868 (V=O), 898 (V—O), 943 (O=V=O), 655 (V—O—V).

## 6. Refinement

Crystal data, data collection and structure refinement details are summarized in Table 3 ▸. H atoms bound to C atoms were positioned geometrically and treated as riding on their parent atoms, with C—H = 0.95 Å and with *U*_iso_(H) = 1.2*U*_eq_(C). H atoms bound to N atoms were discernible in difference-Fourier maps and were refined with N—H bond length restraints of 0.86 (2) Å.

## Supplementary Material

Crystal structure: contains datablock(s) I. DOI: 10.1107/S2056989024005528/wm5720sup1.cif

Structure factors: contains datablock(s) I. DOI: 10.1107/S2056989024005528/wm5720Isup3.hkl

CCDC reference: 2291372

Additional supporting information:  crystallographic information; 3D view; checkCIF report

## Figures and Tables

**Table 1:** Selected bond lengths (Å)

V1—O1	1.6300 (14)	V2—O6	1.6317 (14)
V1—O2	1.6534 (14)	V2—O5	1.6521 (14)
V1—O3	1.8061 (15)	V2—O3	1.8049 (14)
V1—O4^i^	1.8280 (14)	V2—O4	1.8118 (14)

Symmetry code: (i) 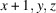.

**Table 2:** Hydrogen-bond geometry (Å, °)

*D*—H⋯*A*	*D*—H	H⋯*A*	*D*⋯*A*	*D*—H⋯*A*
N3*B*—H3*BA*⋯O6	0.88	1.97	2.830	169 (2)
N1*A*—H1*A*⋯O2	0.87	1.85	2.722	174 (2)
N1*B*—H1*B*⋯O5	0.88	1.91	2.774	171 (2)
N3*A*—H3*AA*⋯O1	0.87	1.97	2.837	169 (2)
N2*A*—H2*A*⋯O4^ii^	0.87	1.93	2.798	177 (2)
N2*B*—H2*B*⋯O2^iii^	0.87	2.44	3.123	137 (2)
N2*B*—H2*B*⋯O3^iv^	0.87	2.26	2.960	138 (2)
N3*B*—H3*BB*⋯O3^iv^	0.87	2.18	2.938	145 (2)
N3*B*—H3*BB*⋯O6^v^	0.87	2.41	2.887	115 (2)
N3*A*—H3*AB*⋯O5^ii^	0.88	1.90	2.779	175 (2)

Symmetry codes: (ii) 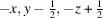; (iii) 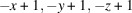; (iv) 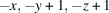; (v) 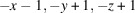.

**Table 3:** Experimental details

Crystal data	
Chemical formula	(C_7_H_8_N_3_)_2_[V_2_O_6_]
*M*_r_	466.21
Crystal system, space group	Monoclinic, *P*2_1_/*c*
Temperature (K)	100
*a*, *b*, *c* (Å)	4.8817 (1), 16.8263 (2), 22.4354 (2)
β (°)	90.675 (1)
*V* (Å^3^)	1842.74 (5)
*Z*	4
Radiation type	Cu *K*α
μ (mm^−1^)	8.93
Crystal size (mm)	0.28 × 0.24 × 0.18

Data collection	
Diffractometer	XtaLAB Synergy, Single source at home/near, HyPix3000
Absorption correction	Multi-scan (*CrysAlis PRO*; Rigaku OD, 2020 ▸)
*T*_min_, *T*_max_	0.349, 1.000
No. of measured, independent and observed [*I* > 2σ(*I*)] reflections	17440, 3540, 3413
*R*_int_	0.037
(sin θ/λ)_max_ (Å^−1^)	0.615

Refinement	
*R*[*F*^2^ > 2σ(*F*^2^)], *wR*(*F*^2^), *S*	0.030, 0.084, 1.07
No. of reflections	3540
No. of parameters	286
No. of restraints	8
H-atom treatment	H atoms treated by a mixture of independent and constrained refinement
Δρ_max_, Δρ_min_ (e Å^−3^)	0.38, −0.44

Computer programs: *CrysAlis PRO* (Rigaku OD, 2020 ▸), *SHELXT* (Sheldrick, 2015*a* ▸), *SHELXL* (Sheldrick, 2015*b* ▸), *Mercury* (Macrae *et al.*, 2020 ▸), *PLATON* (Spek, 2020 ▸) and *publCIF* (Westrip, 2010 ▸).

## supplementary crystallographic information

### catena-Poly[2-aminobenzimidazolium [[dioxidovanadium(V)]-µ-oxido]] Crystal data

**Table:**

(C_7_H_8_N_3_)_2_[V_2_O_6_]	*F*(000) = 944
*M**_r_* = 466.21	*D*_x_ = 1.680 Mg m^−^^3^
Monoclinic, *P*2_1_/*c*	Cu *K*α radiation, λ = 1.54184 Å
*a* = 4.8817 (1) Å	Cell parameters from 11386 reflections
*b* = 16.8263 (2) Å	θ = 3.3–71.2°
*c* = 22.4354 (2) Å	µ = 8.93 mm^−^^1^
β = 90.675 (1)°	*T* = 100 K
*V* = 1842.74 (5) Å^3^	Block, green
*Z* = 4	0.28 × 0.24 × 0.18 mm

### catena-Poly[2-aminobenzimidazolium [[dioxidovanadium(V)]-µ-oxido]] Data collection

**Table:**

XtaLAB Synergy, Single source at home/near, HyPix3000 diffractometer	3413 reflections with *I* > 2σ(*I*)
Detector resolution: 10.0000 pixels mm^-1^	*R*_int_ = 0.037
ω scans	θ_max_ = 71.5°, θ_min_ = 3.3°
Absorption correction: multi-scan (CrysAlisPro; Rigaku OD, 2020)	*h* = −5→5
*T*_min_ = 0.349, *T*_max_ = 1.000	*k* = −20→20
17440 measured reflections	*l* = −27→27
3540 independent reflections	

### catena-Poly[2-aminobenzimidazolium [[dioxidovanadium(V)]-µ-oxido]] Refinement

**Table:**

Refinement on *F*^2^	Hydrogen site location: mixed
Least-squares matrix: full	H atoms treated by a mixture of independent and constrained refinement
*R*[*F*^2^ > 2σ(*F*^2^)] = 0.030	*w* = 1/[σ^2^(*F*_o_^2^) + (0.0486*P*)^2^ + 1.0444*P*] where *P* = (*F*_o_^2^ + 2*F*_c_^2^)/3
*wR*(*F*^2^) = 0.084	(Δ/σ)_max_ = 0.001
*S* = 1.07	Δρ_max_ = 0.38 e Å^−^^3^
3540 reflections	Δρ_min_ = −0.44 e Å^−^^3^
286 parameters	Extinction correction: SHELXL (Sheldrick, 2015b), Fc^*^=kFc[1+0.001xFc^2^λ^3^/sin(2θ)]^-1/4^
8 restraints	Extinction coefficient: 0.00059 (12)

### catena-Poly[2-aminobenzimidazolium [[dioxidovanadium(V)]-µ-oxido]] Special details

**Table:**

Geometry. All esds (except the esd in the dihedral angle between two l.s. planes) are estimated using the full covariance matrix. The cell esds are taken into account individually in the estimation of esds in distances, angles and torsion angles; correlations between esds in cell parameters are only used when they are defined by crystal symmetry. An approximate (isotropic) treatment of cell esds is used for estimating esds involving l.s. planes.

### catena-Poly[2-aminobenzimidazolium [[dioxidovanadium(V)]-µ-oxido]] Fractional atomic coordinates and isotropic or equivalent isotropic displacement parameters (Å^2^)

**Table:**

	*x*	*y*	*z*	*U*_iso_*/*U*_eq_	
V2	−0.28372 (6)	0.46447 (2)	0.35937 (2)	0.01360 (11)	
V1	0.24874 (6)	0.34781 (2)	0.33048 (2)	0.01340 (11)	
O4	−0.5361 (3)	0.42994 (8)	0.30547 (6)	0.0165 (3)	
O6	−0.4298 (3)	0.48306 (8)	0.42291 (6)	0.0194 (3)	
O3	−0.0282 (3)	0.38899 (8)	0.37331 (6)	0.0175 (3)	
O2	0.4310 (3)	0.28954 (8)	0.37509 (6)	0.0182 (3)	
O5	−0.1386 (3)	0.54667 (8)	0.33474 (6)	0.0194 (3)	
O1	0.1365 (3)	0.29770 (8)	0.27302 (6)	0.0213 (3)	
N2B	0.1986 (3)	0.68245 (10)	0.51241 (7)	0.0161 (3)	
N1B	0.0739 (3)	0.64665 (10)	0.42202 (7)	0.0165 (3)	
N1A	0.6212 (4)	0.14345 (10)	0.34384 (7)	0.0175 (3)	
N3B	−0.1562 (4)	0.58715 (10)	0.50284 (7)	0.0190 (3)	
N3A	0.3272 (4)	0.13954 (10)	0.25959 (8)	0.0209 (4)	
N2A	0.6531 (4)	0.03991 (10)	0.28485 (7)	0.0176 (3)	
C2A	0.8201 (4)	0.09402 (11)	0.36893 (9)	0.0170 (4)	
C1B	0.0284 (4)	0.63523 (11)	0.48039 (8)	0.0154 (4)	
C1A	0.5216 (4)	0.10926 (12)	0.29382 (9)	0.0176 (4)	
C2B	0.2814 (4)	0.70255 (11)	0.41573 (8)	0.0158 (4)	
C7B	0.3623 (4)	0.72532 (11)	0.47309 (8)	0.0155 (4)	
C7A	0.8420 (4)	0.02816 (12)	0.33111 (9)	0.0171 (4)	
C3B	0.4005 (4)	0.73529 (12)	0.36553 (9)	0.0201 (4)	
H3B	0.343148	0.720517	0.326468	0.024*	
C6B	0.5667 (4)	0.78067 (12)	0.48278 (9)	0.0181 (4)	
H6B	0.621111	0.796047	0.521905	0.022*	
C3A	0.9760 (4)	0.10065 (12)	0.42048 (9)	0.0212 (4)	
H3A	0.957695	0.144818	0.446506	0.025*	
C5B	0.6896 (4)	0.81295 (12)	0.43252 (9)	0.0210 (4)	
H5B	0.832215	0.850884	0.437509	0.025*	
C4B	0.6082 (4)	0.79089 (12)	0.37508 (9)	0.0221 (4)	
H4B	0.695992	0.814193	0.341798	0.027*	
C4A	1.1607 (5)	0.03984 (13)	0.43252 (10)	0.0248 (5)	
H4A	1.271173	0.042284	0.467625	0.030*	
C6A	1.0281 (4)	−0.03202 (12)	0.34269 (10)	0.0213 (4)	
H6A	1.045994	−0.076194	0.316655	0.026*	
C5A	1.1874 (4)	−0.02505 (13)	0.39392 (10)	0.0242 (4)	
H5A	1.317900	−0.065244	0.403102	0.029*	
H3BA	−0.259 (4)	0.5555 (12)	0.4812 (9)	0.023 (6)*	
H2B	0.217 (5)	0.6788 (16)	0.5508 (5)	0.026 (7)*	
H3AA	0.251 (5)	0.1857 (9)	0.2664 (12)	0.031 (7)*	
H2A	0.619 (5)	0.0069 (13)	0.2558 (9)	0.029 (7)*	
H3BB	−0.160 (5)	0.5797 (17)	0.5413 (5)	0.034 (7)*	
H3AB	0.263 (5)	0.1129 (14)	0.2288 (8)	0.027 (7)*	
H1B	−0.004 (5)	0.6193 (15)	0.3933 (9)	0.036 (7)*	
H1A	0.566 (5)	0.1897 (9)	0.3563 (12)	0.035 (7)*	

### catena-Poly[2-aminobenzimidazolium [[dioxidovanadium(V)]-µ-oxido]] Atomic displacement parameters (Å^2^)

**Table:**

	*U*^11^	*U*^22^	*U*^33^	*U*^12^	*U*^13^	*U*^23^
V2	0.01470 (19)	0.01364 (18)	0.01242 (17)	−0.00046 (11)	−0.00200 (12)	0.00155 (11)
V1	0.01604 (19)	0.01193 (17)	0.01219 (17)	−0.00065 (11)	−0.00225 (12)	0.00014 (11)
O4	0.0192 (7)	0.0153 (7)	0.0150 (6)	−0.0008 (5)	−0.0029 (5)	0.0023 (5)
O6	0.0199 (7)	0.0207 (7)	0.0174 (7)	−0.0005 (5)	0.0004 (5)	0.0001 (5)
O3	0.0172 (7)	0.0192 (7)	0.0160 (6)	0.0011 (5)	−0.0027 (5)	0.0011 (5)
O2	0.0226 (7)	0.0155 (7)	0.0166 (6)	0.0018 (5)	−0.0009 (5)	0.0001 (5)
O5	0.0227 (7)	0.0195 (7)	0.0159 (7)	−0.0033 (5)	−0.0033 (5)	0.0026 (5)
O1	0.0268 (8)	0.0190 (7)	0.0181 (7)	−0.0018 (6)	−0.0043 (6)	−0.0015 (5)
N2B	0.0208 (8)	0.0151 (8)	0.0124 (7)	−0.0007 (6)	−0.0026 (6)	0.0011 (6)
N1B	0.0185 (8)	0.0177 (8)	0.0133 (8)	−0.0022 (6)	−0.0008 (6)	−0.0012 (6)
N1A	0.0224 (9)	0.0134 (8)	0.0165 (8)	0.0013 (6)	−0.0017 (7)	−0.0042 (6)
N3B	0.0218 (9)	0.0186 (8)	0.0165 (8)	−0.0027 (7)	0.0006 (7)	0.0002 (7)
N3A	0.0291 (10)	0.0164 (8)	0.0171 (8)	0.0017 (7)	−0.0044 (7)	−0.0037 (7)
N2A	0.0225 (9)	0.0141 (8)	0.0162 (8)	−0.0013 (6)	−0.0004 (7)	−0.0035 (6)
C2A	0.0166 (9)	0.0150 (9)	0.0195 (9)	−0.0016 (7)	0.0008 (7)	0.0005 (7)
C1B	0.0174 (9)	0.0133 (9)	0.0155 (9)	0.0042 (7)	−0.0017 (7)	−0.0001 (7)
C1A	0.0206 (10)	0.0164 (9)	0.0159 (9)	−0.0030 (7)	0.0022 (7)	−0.0001 (7)
C2B	0.0166 (9)	0.0141 (9)	0.0168 (9)	0.0016 (7)	−0.0008 (7)	−0.0005 (7)
C7B	0.0165 (9)	0.0147 (9)	0.0152 (9)	0.0028 (7)	0.0001 (7)	0.0011 (7)
C7A	0.0184 (10)	0.0168 (9)	0.0161 (9)	−0.0024 (7)	0.0038 (7)	−0.0006 (7)
C3B	0.0258 (11)	0.0198 (10)	0.0148 (9)	0.0014 (8)	0.0022 (8)	0.0003 (7)
C6B	0.0181 (10)	0.0168 (9)	0.0194 (10)	0.0015 (7)	−0.0039 (7)	−0.0003 (7)
C3A	0.0212 (10)	0.0203 (10)	0.0220 (10)	−0.0012 (8)	−0.0013 (8)	−0.0035 (8)
C5B	0.0171 (10)	0.0178 (10)	0.0282 (10)	−0.0004 (8)	−0.0006 (8)	0.0020 (8)
C4B	0.0242 (11)	0.0203 (10)	0.0221 (10)	0.0015 (8)	0.0060 (8)	0.0043 (8)
C4A	0.0223 (11)	0.0260 (11)	0.0261 (11)	−0.0008 (8)	−0.0047 (9)	0.0012 (8)
C6A	0.0220 (10)	0.0164 (10)	0.0256 (10)	0.0000 (7)	0.0043 (8)	−0.0007 (8)
C5A	0.0202 (10)	0.0199 (10)	0.0326 (12)	0.0027 (8)	0.0008 (9)	0.0053 (9)

### catena-Poly[2-aminobenzimidazolium [[dioxidovanadium(V)]-µ-oxido]] Geometric parameters (Å, º)

**Table:**

V1—O1	1.6300 (14)	N2A—C1A	1.348 (3)
V1—O2	1.6534 (14)	N2A—C7A	1.394 (3)
V1—O3	1.8061 (15)	N2A—H2A	0.871 (10)
V1—O4^i^	1.8280 (14)	C2A—C3A	1.381 (3)
V2—O6	1.6317 (14)	C2A—C7A	1.401 (3)
V2—O5	1.6521 (14)	C2B—C3B	1.387 (3)
V2—O3	1.8049 (14)	C2B—C7B	1.395 (3)
V2—O4	1.8118 (14)	C7B—C6B	1.380 (3)
V2—V1^ii^	3.0736 (4)	C7A—C6A	1.383 (3)
N2B—C1B	1.351 (3)	C3B—C4B	1.394 (3)
N2B—C7B	1.398 (2)	C3B—H3B	0.9500
N2B—H2B	0.866 (10)	C6B—C5B	1.394 (3)
N1B—C1B	1.345 (3)	C6B—H6B	0.9500
N1B—C2B	1.391 (3)	C3A—C4A	1.388 (3)
N1B—H1B	0.873 (10)	C3A—H3A	0.9500
N1A—C1A	1.347 (3)	C5B—C4B	1.394 (3)
N1A—C2A	1.393 (3)	C5B—H5B	0.9500
N1A—H1A	0.871 (10)	C4B—H4B	0.9500
N3B—C1B	1.315 (3)	C4A—C5A	1.401 (3)
N3B—H3BA	0.874 (10)	C4A—H4A	0.9500
N3B—H3BB	0.873 (10)	C6A—C5A	1.385 (3)
N3A—C1A	1.316 (3)	C6A—H6A	0.9500
N3A—H3AA	0.875 (10)	C5A—H5A	0.9500
N3A—H3AB	0.877 (10)		
			
O6—V2—O5	108.98 (7)	N1B—C1B—N2B	109.04 (17)
O6—V2—O3	106.96 (7)	N3A—C1A—N1A	124.85 (18)
O5—V2—O3	110.42 (7)	N3A—C1A—N2A	126.10 (18)
O6—V2—O4	110.11 (7)	N1A—C1A—N2A	109.05 (17)
O5—V2—O4	109.64 (7)	C3B—C2B—N1B	131.57 (18)
O3—V2—O4	110.68 (6)	C3B—C2B—C7B	121.52 (18)
O1—V1—O2	110.21 (7)	N1B—C2B—C7B	106.90 (16)
O1—V1—O3	111.87 (7)	C6B—C7B—C2B	121.79 (18)
O2—V1—O3	107.84 (6)	C6B—C7B—N2B	131.79 (18)
O1—V1—O4^i^	109.71 (7)	C2B—C7B—N2B	106.42 (16)
O2—V1—O4^i^	109.10 (7)	C6A—C7A—N2A	132.15 (18)
O3—V1—O4^i^	108.04 (6)	C6A—C7A—C2A	121.32 (19)
V2—O4—V1^ii^	115.22 (7)	N2A—C7A—C2A	106.53 (17)
V2—O3—V1	134.26 (8)	C2B—C3B—C4B	116.91 (19)
C1B—N2B—C7B	108.71 (16)	C2B—C3B—H3B	121.5
C1B—N2B—H2B	122.9 (18)	C4B—C3B—H3B	121.5
C7B—N2B—H2B	127.5 (18)	C7B—C6B—C5B	116.93 (18)
C1B—N1B—C2B	108.93 (16)	C7B—C6B—H6B	121.5
C1B—N1B—H1B	124.6 (19)	C5B—C6B—H6B	121.5
C2B—N1B—H1B	126.2 (19)	C2A—C3A—C4A	116.97 (19)
C1A—N1A—C2A	108.96 (16)	C2A—C3A—H3A	121.5
C1A—N1A—H1A	122.5 (19)	C4A—C3A—H3A	121.5
C2A—N1A—H1A	128.5 (19)	C6B—C5B—C4B	121.54 (19)
C1B—N3B—H3BA	123.6 (16)	C6B—C5B—H5B	119.2
C1B—N3B—H3BB	119.4 (19)	C4B—C5B—H5B	119.2
H3BA—N3B—H3BB	116 (3)	C3B—C4B—C5B	121.30 (18)
C1A—N3A—H3AA	123.1 (18)	C3B—C4B—H4B	119.4
C1A—N3A—H3AB	120.6 (18)	C5B—C4B—H4B	119.4
H3AA—N3A—H3AB	116 (2)	C3A—C4A—C5A	121.3 (2)
C1A—N2A—C7A	108.88 (16)	C3A—C4A—H4A	119.4
C1A—N2A—H2A	125.2 (18)	C5A—C4A—H4A	119.4
C7A—N2A—H2A	125.9 (18)	C7A—C6A—C5A	117.07 (19)
C3A—C2A—N1A	131.68 (18)	C7A—C6A—H6A	121.5
C3A—C2A—C7A	121.74 (19)	C5A—C6A—H6A	121.5
N1A—C2A—C7A	106.57 (17)	C6A—C5A—C4A	121.6 (2)
N3B—C1B—N1B	125.63 (18)	C6A—C5A—H5A	119.2
N3B—C1B—N2B	125.31 (18)	C4A—C5A—H5A	119.2
			
O6—V2—O4—V1^ii^	−51.54 (10)	C3B—C2B—C7B—N2B	178.79 (18)
O5—V2—O4—V1^ii^	−171.43 (7)	N1B—C2B—C7B—N2B	−0.4 (2)
O3—V2—O4—V1^ii^	66.52 (9)	C1B—N2B—C7B—C6B	−179.8 (2)
O6—V2—O3—V1	−168.95 (10)	C1B—N2B—C7B—C2B	0.6 (2)
O5—V2—O3—V1	−50.50 (13)	C1A—N2A—C7A—C6A	−179.1 (2)
O4—V2—O3—V1	71.09 (12)	C1A—N2A—C7A—C2A	−0.1 (2)
V1^ii^—V2—O3—V1	100.81 (10)	C3A—C2A—C7A—C6A	−2.3 (3)
O1—V1—O3—V2	−67.14 (13)	N1A—C2A—C7A—C6A	178.54 (17)
O2—V1—O3—V2	171.52 (10)	C3A—C2A—C7A—N2A	178.57 (17)
O4^i^—V1—O3—V2	53.72 (12)	N1A—C2A—C7A—N2A	−0.6 (2)
V2^i^—V1—O3—V2	86.44 (11)	N1B—C2B—C3B—C4B	−179.92 (19)
C1A—N1A—C2A—C3A	−177.9 (2)	C7B—C2B—C3B—C4B	1.2 (3)
C1A—N1A—C2A—C7A	1.1 (2)	C2B—C7B—C6B—C5B	−0.1 (3)
C2B—N1B—C1B—N3B	179.08 (18)	N2B—C7B—C6B—C5B	−179.57 (19)
C2B—N1B—C1B—N2B	0.5 (2)	N1A—C2A—C3A—C4A	−179.6 (2)
C7B—N2B—C1B—N3B	−179.32 (18)	C7A—C2A—C3A—C4A	1.5 (3)
C7B—N2B—C1B—N1B	−0.7 (2)	C7B—C6B—C5B—C4B	0.6 (3)
C2A—N1A—C1A—N3A	178.88 (18)	C2B—C3B—C4B—C5B	−0.7 (3)
C2A—N1A—C1A—N2A	−1.2 (2)	C6B—C5B—C4B—C3B	−0.2 (3)
C7A—N2A—C1A—N3A	−179.26 (19)	C2A—C3A—C4A—C5A	0.2 (3)
C7A—N2A—C1A—N1A	0.8 (2)	N2A—C7A—C6A—C5A	−179.8 (2)
C1B—N1B—C2B—C3B	−179.1 (2)	C2A—C7A—C6A—C5A	1.3 (3)
C1B—N1B—C2B—C7B	0.0 (2)	C7A—C6A—C5A—C4A	0.3 (3)
C3B—C2B—C7B—C6B	−0.8 (3)	C3A—C4A—C5A—C6A	−1.0 (3)
N1B—C2B—C7B—C6B	−179.98 (17)		

Symmetry codes: (i) *x*+1, *y*, *z*; (ii) *x*−1, *y*, *z*.

### catena-Poly[2-aminobenzimidazolium [[dioxidovanadium(V)]-µ-oxido]] Hydrogen-bond geometry (Å, º)

**Table:**

*D*—H···*A*	*D*—H	H···*A*	*D*···*A*	*D*—H···*A*
N3*B*—H3*BA*···O6	0.88	1.97	2.830	169 (2)
N1*A*—H1*A*···O2	0.87	1.85	2.722	174 (2)
N1*B*—H1*B*···O5	0.88	1.91	2.774	171 (2)
N3*A*—H3*AA*···O1	0.87	1.97	2.837	169 (2)
N2*A*—H2*A*···O4^iii^	0.87	1.93	2.798	177 (2)
N2*B*—H2*B*···O2^iv^	0.87	2.44	3.123	137 (2)
N2*B*—H2*B*···O3^v^	0.87	2.26	2.960	138 (2)
N3*B*—H3*BB*···O3^v^	0.87	2.18	2.938	145 (2)
N3*B*—H3*BB*···O6^vi^	0.87	2.41	2.887	115 (2)
N3*A*—H3*AB*···O5^iii^	0.88	1.90	2.779	175 (2)

Symmetry codes: (iii) −*x*, *y*−1/2, −*z*+1/2; (iv) −*x*+1, −*y*+1, −*z*+1; (v) −*x*, −*y*+1, −*z*+1; (vi) −*x*−1, −*y*+1, −*z*+1.
